# Effect of Segmented Optical Axial Length on the Performance of New-Generation Intraocular Lens Power Calculation Formulas in Extremely Long Eyes

**DOI:** 10.3390/jcm12226959

**Published:** 2023-11-07

**Authors:** So Goto, Naoyuki Maeda, Kota Uehara, Keiko Ogawa, Maki Matsumaru, Saori Sugiyama, Kazuhiko Ohnuma, Tjundewo Lawu, Toru Noda

**Affiliations:** 1 Department of Ophthalmology, Osaka University Graduate School of Medicine, Yamadaoka 2-2, Suita 565-0871, Osaka, Japan; nmaeda@ophthal.med.osaka-u.ac.jp; 2 Department of Ophthalmology, National Hospital Organization, Tokyo Medical Center, 2-5-1 Higashigaoka, Meguro-ku, Tokyo 152-8902, Japan; kouta3121@icloud.com (K.U.); keiko.ogawa@kankakuki.jp (K.O.); makimatsumaru@gmail.com (M.M.); suusuu.n.n.zzz@gmail.com (S.S.); mieemi@ga2.so-net.ne.jp (T.N.); 3 Herbert Wertheim School of Optometry and Vision Science, University of California, Berkeley, CA 94720, USA; 4 Laboratorio de Lente Verde, 98-1 Nozomino, Sodegaura 299-0251, Chiba, Japan; kazuhiko.oonuma625@gmail.com; 5 VO, 3-10-20 Honcho, Toda 335-0023, Saitama, Japan; tjundewo@visualoptics.co

**Keywords:** segmented axial length, extremely long eye, swept-source optical coherence tomography-based biometer, new-generation IOL power calculation formulas

## Abstract

Purpose: To evaluate the performance of traditional vergence formulas with segmented axial length (AL) compared to traditional composite AL in extremely long eyes, and to determine whether the segmented AL can be extended to the new-generation formulas, including the Barrett Universal II, Emmetropia Verifying Optical 2.0 (EVO2), Hill-RBF 3.0 (Hill3), Kane, and Ladas Super formula (LSF) formulas in extremely long eyes. Setting: National Hospital. Organization, Tokyo Medical Center, Japan. Design: Retrospective case series. Methods: Consecutive patients who underwent uncomplicated cataract surgery implanted with a three-piece intraocular lens between December 2015 and March 2021 were retrospectively reviewed. The composite AL was measured with a swept-source optical coherence tomography (SS-OCT) biometer using a mean refractive index. The segmented AL was calculated by summing the geometric lengths of the ocular segments (cornea, aqueous, lens, and vitreous) using multiple specific refractive indices based on the data obtained by the SS-OCT-based biometer. When refraction was measured at three months postoperatively, the median absolute errors (MedAEs) were calculated with two ALs for each formula. Results: The study included 31 eyes of 22 patients. The segmented AL (30.45 ± 1.23 mm) was significantly shorter than the composite AL (30.71 ± 1.28 mm, *p* < 0.001). The MedAEs were significantly reduced when using segmented AL for SRK/T, Haigis, Hill3, and LSF, compared to those obtained using composite AL (0.38 vs. 0.62, 0.48 vs. 0.79, 0.50 vs. 0.90, 0.34 vs. 0.61, *p* < 0.001 for all formulas, respectively). On the contrary, the MedAE obtained by Kane with segmented AL was significantly worse compared to the one with composite AL (0.35 vs. 0.27, *p* = 0.03). Conclusion: In extremely high myopic eyes, the segmented AL improves the performance of SRK/T, Haigis, Hill3, and LSF formulas compared to the composite AL, while the segmented AL worsens the prediction accuracy of the Kane formula.

## 1. Introduction

In the year 2050, it is estimated that half of the global population will be affected by myopia, with 10% of those having high myopia [1]. High myopia was associated with an increased incidence of cataracts [2,3]. Although the refractive outcomes after cataract surgery have been improved with the evolution of intraocular lens (IOL) power calculation formulas, the prediction accuracy for high myopia is still challenging [4,5,6]. Traditional IOL power calculation formulas tend to predict hyperopic refractive errors in longer eyes. Despite the introduction of new-generation formulas, challenges in predicting postoperative refraction accuracy remain. Furthermore, challenges remain with regard to highly myopic eyes [7].

The axial length (AL) measurement is a fundamental parameter in the calculation of IOL power. Theoretically, optical AL is available in two different theories: the use of a single group refractive index for the entire eye (composite AL) and the use of multiple specific refractive indices for summing the geometric lengths of the ocular segments (segmented AL) [8,9,10]. Segmented AL (also known as sum-of-segments AL) measures, via an optical biometer, improved refractive prediction accuracy for vergence formulas [8,9,10]. Optical biometry has been a major contributor to the improved accuracy of AL measurements [11,12,13]. Newly introduced optical biometers based on swept-source optical coherence tomography (SS-OCT) have been shown to generate repeatable and reproducible measurements [14,15]. Although the SS-OCT biometer has reported the efficacy of segmented AL for the existing formula, the impact of segmented AL measurement on the latest IOL formulas for extremely long eyes remains uncertain.

The purpose of this study was to evaluate the performance of new-generation IOL formulas, including the Barrett Universal II (BUII) [16], Emmetropia Verifying Optical 2.0 (EVO2), Hill-RBF 3.0 (Hill3) [17], Kane [18], and Ladas Super formula (LSF) [19] formulas, with segmented AL in comparison to the traditional composite AL in extremely long eyes.

## 2. Materials and Methods

In this retrospective case-series study, we reviewed consecutive cases of patients with cataracts who underwent phacoemulsification with the in-the-bag IOL implantation of an aspheric acrylic 3-piece IOL (AN6MA, Kowa, Nagoya, Japan) at the National Hospital Organization, Tokyo Medical Center, from December 2015 to March 2021. This study received approval from the Institutional Review Board of the National Hospital Organization, Tokyo Medical Center (R18-161), and was conducted in accordance with the tenets of the Declaration of Helsinki. All patients provided written informed consent.

The inclusion criteria were set as eyes with an extremely long AL of 28.5 mm or more preoperatively. The exclusion criteria for the study were as follows: corrected distance visual acuity (CDVA) after cataract surgery less than or equal to 20/40; preoperative or post-operative astigmatism greater than 4.0 diopters (D); history of ocular surgery and/or ocular trauma; the presence of a significant ocular comorbidity (e.g., myopic maculopathy, ocular surface diseases, corneal opacity, keratoconus, pterygium, or pseudoexfoliation syndrome); unreliable or undetectable preoperative biometry measurements; a history of intra- or post-operative complications; or participants unable to provide consent for contact lens removal one month prior to the preoperative assessment.

### 2.1. Patient Examinations

AL measurements were obtained preoperatively using SS-OCT with a single refractive index for an entire eye (OA-2000, Ver.4F; Tomey Corporation, Nagoya, Japan). All patients underwent routine preoperative and three-month post-operative ophthalmic examinations, including a CDVA measurement using a Landolt C chart at 5 m, slit-lamp examination, keratometry, intraocular pressure measurement, and fundoscopy. In cases where participants utilized contact lenses, their use was discontinued one month prior to the preoperative measurement. Cataract surgery was performed via a temporal self-sealing corneal incision, followed by phacoemulsification and in-the-bag IOL implantation. All surgical procedures were performed under topical anesthesia by the same experienced surgeon (T.N.).

### 2.2. Calculation of Segmented Axial Length

An SS-OCT-based optical biometer (OA-2000) measures the optical path length of the whole eye using a mean group refractive index of 1.3496 for a phakic eye. The segmented AL was calculated by summing the segmented parts of the eye with individual refractive indices of 1.3837 for the cornea, 1.3695 for the anterior chamber depth (ACD), 1.3394 for the aqueous and vitreous, and 1.4051 for a crystalline lens; all these indices were provided from the manufacturer which offers the biometer. The SS-OCT-based biometer (OA-2000) directly measured and displayed the composite AL, central corneal thickness (CCT), ACD, and lens thickness (LT). The vitreous chamber depth (VCD) was calculated backward using composite AL, ACD, CCT, and LT. In the following equation (Equation (1))

Segmented AL = CCT/1000 + AQD + LT + VCD + Offset = CCT/1000 + (1.3695 × ACD − 1.3837 × CCT/1000)/1.3394 + LT + [1.3496 × (0.9573 × Composite AL + 1.3304) − (1.3695 × ACD − 1.3837 × CCT/1000) − 1.4051 × LT − 1.3837 × CCT/1000]/1.3394 − 0.27(1)

AL indicates axial length (mm), ACD is the anterior chamber depth (mm), AQD is the aqueous depth (mm), CCT is the central corneal thickness (µm), LT is the crystalline lens thickness (mm), and VCD is the vitreous chamber depth (mm). Refractive indices (RIs) of the individual tissue were used during the calculation.

### 2.3. IOL Power Calculation and Data Collection

The BUII (V1.05), EVO2, Kane, Hill3, and LSF formulas (accessed between 13 February 2022 and 3 March 2022) were calculated via their respective websites with an A-constant of 117.4, which was provided by the manufacture (KOWA). The SRK/T and Haigis formulas were calculated via an Excel spreadsheet (Microsoft Office 2019; Microsoft Corporation, Redmond, WA, USA) with an A-constant of 117.4. The prediction error was obtained as the postoperative manifest refraction minus the predicted refractive values calculated by four formulas using the implanted IOL power. The mean numerical prediction error (MPE), mean absolute refractive prediction error (MAE), and median absolute prediction error (MedAE) were also calculated for each formula.

### 2.4. Statistical Analysis

Statistical analyses were performed with JMP Pro version 14.0.0 (SAS Institute Inc., Cary, NC, USA). The composite and segmented ALs were compared by using the paired *t*-test. The Bland–Altman limits-of-agreement method was used to assess the agreement in two ALs. Depending on whether the data were normally distributed or not, either the paired t-test or Wilcoxon signed-rank test was used to determine whether the MPEs were significantly different from zero. The Wilcoxon rank sum test was used to compare the MedAE values calculated with the segmented AL and composite AL for each IOL power formula. The McNemar test was performed to evaluate if the percentages of eyes within 0.5 D and 1.0 D of refractive prediction errors were significantly different with the two ALs for each IOL power formula. The sample size was calculated to detect a difference in the median absolute error of 0.5 D between two groups; with a significance level of 5%, a statistical power of 80%, and assuming standard deviation (SD) to 0.45 D, 28 eyes were required. *p* values were adjusted for multiple comparisons, and an adjusted *p* value less than 0.05 was considered statistically significant.

## 3. Results

The medical records of 31 eyes from 22 patients (16 eyes from 10 females, 15 eyes from 12 males) were retrospectively reviewed. The subjects had undergone routine cataract surgery at a mean age of 69.6 ± 10.0 (range 48 to 91) years. The mean IOL power was 2.0 ± 1.8 D (range −3.0 to +5.0 D).

**Figure 1:**
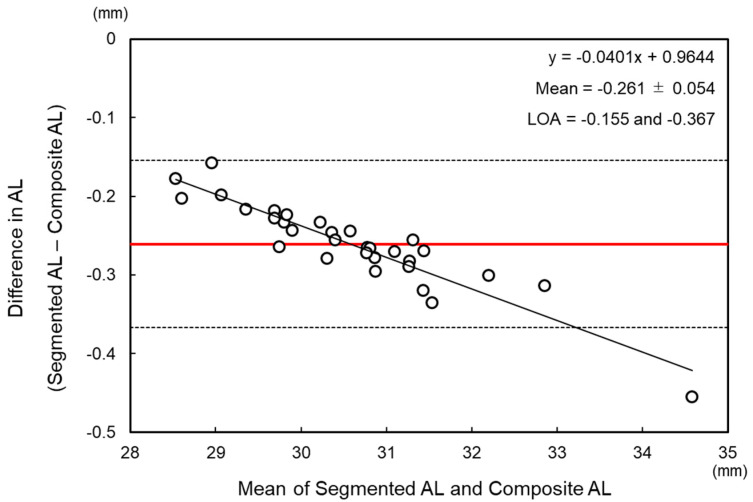
Bland–Altman plots of composite and segmented axial lengths. The limits of agreement (LOA) were set at ±1.96 × standard deviation. The thin solid black line indicates zero, the thick solid red line indicates the mean, and the dashed lines indicate the upper and lower limits of agreement. AL = axial length, RI = refractive index.

The mean preoperative measurement values are shown in Table 1. The segmented AL (30.45 ± 1.23 mm) was significantly shorter than the composite AL (30.71 ± 1.28 mm, *p* < 0.001). The Bland–Altman analysis revealed a negative proportional bias between the segmented AL and composite AL with the mean difference of −0.261 ± 0.054 mm and 95% limits of agreement of −0.367 to −0.155 mm (Figure 1).

**Figure 2:**
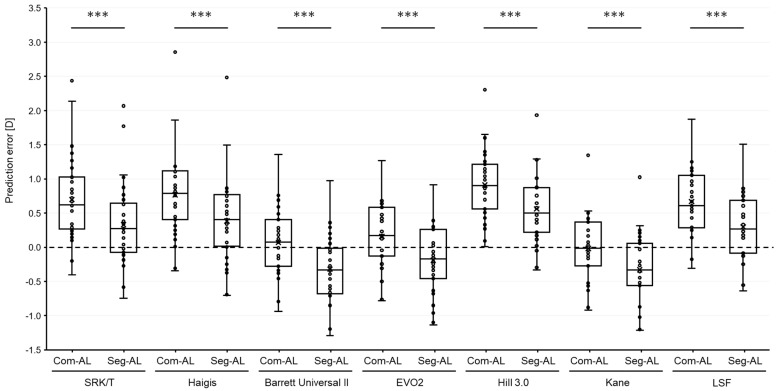
Box plots illustrating the numerical prediction errors in refraction with intraocular lens calculation formulas based on each axial length obtained using two different methods: (1) Com-AL, the traditional composite axial length displayed by the SS-OCT biometer; and (2) Seg-AL, the segmented axial length calculated using data from the SS-OCT biometer. The dashed line indicates a 0 value, and white circles mean outliers. D = diopters, EVO2 = Emmetropia Verifying Optical 2.0, Hill3.0 = Hill-RBF 3.0, LSF = Ladas Super formula. *** *p* < 0.001.

**Figure 3:**
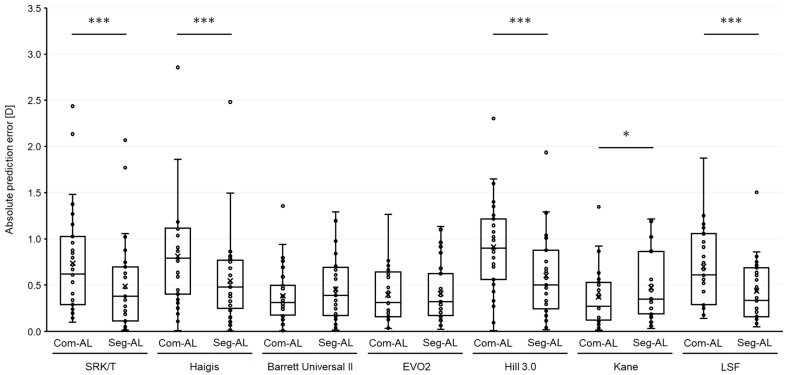
Box plots showing the absolute prediction errors in refraction with intraocular lens calculation formulas based on each axial length obtained by two different methods: (1) Com-AL, the traditional composite axial length displayed by the SS-OCT biometer; and (2) Seg-AL, the segmented axial length calculated using data from the SS-OCT biometer. D = diopters, EVO2 = Emmetropia Verifying Optical 2.0, Hill3.0 = Hill-RBF 3.0, LSF = Ladas Super formula. White circles indicate outliers. * *p* < 0.05, *** *p* < 0.001.

The results of each formula are summarized in Table 2. The MPEs of each AL showed significant differences in all formulas (*p* < 0.0001, Figure 2). MPEs calculated with segmented AL showed a myopic shift in all formulas compared to ones with com AL. The scatter plots illustrating the prediction errors, when comparing the composite AL to segmented AL across various formulas, consistently revealed a strong correlation for all examined formulas (Supplementary Figure S1). The MedAEs obtained using the SRK/T, Haigis, Hill3, and LSF formulas with the segmented AL were significantly lower compared to ones with the composite AL (*p* < 0.0001, respectively, Figure 3). On the contrary, the MedAE obtained using the Kane formula with composite AL was significantly lower compared to that with segmented AL (*p* = 0.03). The MedAEs obtained using the BUII and EVO2 formulas with segmented AL showed no significant differences compared to those with composite AL (*p* = 0.12 and 0.62, respectively).

**Figure 4:**
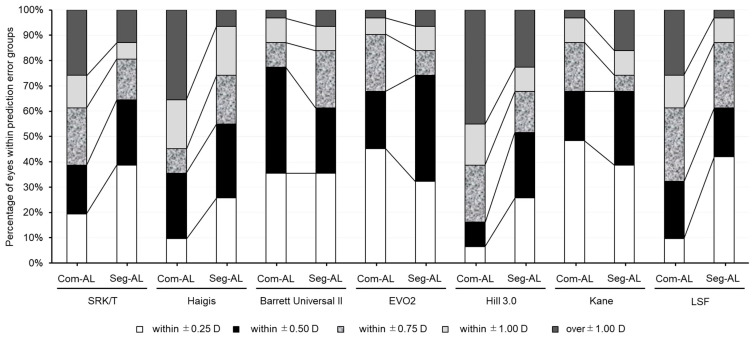
Stacked histogram showing percentage of eyes within ±0.25 diopter (D), ±0.50 D, ±0.75 D, ±1.0 D, and >1.0 D range of prediction error. Axial length measurements were calculated with two different methods: (1) Com-AL, the traditional composite axial length displayed by the SS-OCT biometer; and (2) Seg-AL, the segmented axial length calculated using data from the SS-OCT biometer. EVO2 = Emmetropia Verifying Optical 2.0, Hill3.0 = Hill-RBF 3.0, LSF = Ladas Super formula.

The segmented AL produced a greater percentage of eyes within 0.5 D of error with the Haigis and Hill3 formulas (*p* = 0.035 and 0.008, respectively) and a greater percentage of eyes within 1.0 D of error with the SRK/T, Haigis, Hill3, and LSF formulas (*p* = 0.025, 0.046, 0.014, and 0.046, respectively; Table 2 and Figure 4).

## 4. Discussion

The precise measurement of AL is essential in determining the appropriate IOL power in cataract surgery [12,13]. This retrospective study aimed to evaluate the impact of the segmented AL on IOL power calculation and compare the performance of various IOL formulas between segmented AL and composite AL in extremely long eyes. For eyes with composite AL greater than 28.5 mm, the segmented AL was significantly shorter than that of composite AL with the difference range from −0.45 mm to −0.16 mm. The Bland–Altman analysis revealed a negative proportional bias between the two ALs, indicating that the difference between the two measurements increased as the AL increased. Consequently, the segmented AL demonstrated a myopic shift of refractive prediction errors in all IOL formulas compared to composite AL.

In long eyes, a number of IOL power calculation formulas have been reported to exhibit varying degrees of hyperopic prediction error [4,5,20,21]. According to the findings from the meta-analysis, the Olsen, Kane, and EVO formulas seemingly demonstrate superior predictive precision for longer eyes. Nonetheless, there remains uncertainty regarding this point, which should be validated through extensive multi-center, registry-based studies [22]. In this study, the segmented AL demonstrated improved prediction accuracy for the SRK/T, Haigis, Hill3, and LSF formulas, while it negatively affected the prediction accuracy of the Kane formula. No significant differences were observed for the BUII and EVO2 formulas. Furthermore, the segmented AL demonstrated a higher proportion of eyes within 0.5 D of error with the Haigis and Hill3 formulas and within 1.0 D of error with the SRK/T, Haigis, Hill3, and LSF formulas. These findings suggest that the segmented AL may provide more accurate IOL power calculations using certain formulas, although this was not a universal trend across all formulas assessed.

The outcomes for the SRK/T and Haigis formulas were consistent with previous reports, showing an improvement in the hyperopic shift for eyes with long AL when using the segmented AL [8,9,10]. In contrast, for the latest Hill3 formula, there are limited reports on its performance in long eyes, but hyperopic shifts in prediction error for the Hill3 formula have been reported in eyes with ALs over 28 mm [23,24]. In the current study, the outcome of the Hill3 formula was improved with the use of segmented AL. As the concept of the LSF formula integrates existing formulas, the improvement in the prediction error observed with segmented AL for the LSF formula might be due to the incorporation of characteristics from existing formulas that illustrate a hyperopic shift in prediction error in long eyes [25].

Although the new-generation formulas, BU2 and EVO2 [25], which are presumably optimized for composite AL, showed a minor hyperopic shift in prediction error, the myopic shift derived by using segmented AL serves to displace the slight hyperopic prediction error observed in extremely long eyes towards the myopic direction, yielding a myopic shift above zero. However, this does not give rise to a significant distinction between the magnitudes of hyperopic and myopic shifts when assessed in terms of absolute values of prediction error. Regarding the Kane formula, which has great predictability even in extremely long eyes [7], the utilization of segmented AL led to an excessive myopic shift and deteriorated the prediction accuracy in terms of absolute error.

These results indicate that while segmented AL can improve the prediction accuracy of the existing vergence formulas and Hill3 formula, prudence is warranted when incorporating it with new-generation formulas. Specifically, its application with the Kane formula should be used with caution.

The current study has several limitations. Firstly, the retrospective design might have introduced potential biases in data collection and patient selection. Secondly, the sample size was relatively small, and bilateral eyes were included due to the infrequency of extremely long eyes, potentially leading to data compounding. Additionally, the study only incorporated only cases involving a three-piece IOL which might have a slightly unstable postoperative IOL position. Future prospective studies with larger sample sizes and diverse populations are required to validate our findings and further explore the impact of segmented AL on various IOL power calculation formulas. In this study, lens constant optimization, which is recommended for its ability to reduce myopic or hyperopic prediction errors [26,27], was not employed, as this optimization is only applicable to four open-source formulas: Haigis, Holladay 1, Hoffer Q, and SRK/T, limiting its feasibility for recently developed and online formulas. Recently, the heteroscedasticity test has been utilized to compare the accuracy of IOL formulas [28,29]. However, this test is generally recommended for studies encompassing more than 300 cases. Given the relatively small number of cases in this study, the heteroscedasticity test did not detect any significant differences.

In conclusion, our study demonstrates that the use of segmented AL in extremely long eyes can improve the prediction accuracy of IOL power calculation formulas, particularly SRK/T, Haigis, Hill3, and LSF. However, it is crucial to note that the predictive accuracy of the Kane formula might be compromised with the use of segmented AL. This emphasizes the significance of considering multiple specific refractive indices in AL measurements, reinforcing the need for precision in IOL power calculations for extremely long eyes.

## Figures and Tables

**Table 1:** Demographic and ocular characteristics.

	Mean ± SD	Min	Max
Age (years old)	69.6 ± 10.0	48	91
Gender: female (%)	45		
Central corneal thickness (mm)	524 ± 38	445	577
Anterior chamber depth (mm)	3.57 ± 0.36	2.51	4.2
Lens thickness (mm)	4.62 ± 0.43	3.71	5.65
Composite axial length (mm)	30.71 ± 1.28	28.62	34.81
Segmented axial length (mm)	30.45 ± 1.23	28.44	34.36
Implanted IOL power (diopter)	2.0 ± 1.8	−3.0	5.0

IOL: intraocular lens, SD: standard deviation.

**Table 2:** Refraction prediction errors of the various formulas based on two axial lengths.

	MPE	SD	MAE	MedAE	Maximum	±0.5 (%)	±1.0 (%)
SRK/T-Com AL	0.698	0.613	0.737 *	0.619	2.44	38.7	74.2 *
SRK/T-Seg AL	0.329	0.610	0.489	0.379	2.067	64.5	87.1 *
Haigis-Com AL	0.768	0.620	0.810	0.791	2.86	35.5 *	64.5 *
Haigis-Seg AL	0.385	0.625	0.546	0.481	2.48	54.8 *	93.5 *
BUII-Com AL	0.076	0.472	0.371	0.300	1.355	77.4	96.8
BUII-Seg AL	−0.319	0.475	0.457	0.390	0.975	61.3	93.5
EVO2-Com AL	0.160	0.469	0.396	0.310	1.265	67.7	96.8
EVO2-Seg AL	−0.193	0.480	0.409	0.320	0.915	74.2	93.5
Hill3-Com AL	0.881	0.473	0.911	0.900	2.305	16.1 *	58.1 *
Hill3-Seg AL	0.559	0.491	0.603	0.500	1.935	51.6 *	77.4 *
Kane-Com AL	−0.018	0.492	0.370	0.270	1.35	67.7	96.8
Kane-Seg AL	−0.330	0.503	0.471	0.350	1.03	67.7	83.9
LSF-Com AL	0.447	0.762	0.699	0.610	1.875	32.3	74.2 *
LSF-Seg AL	0.298	0.461	0.437	0.335	1.505	61.3	96.8 *

BUII: Barrett Universal II, Com AL: composite axial length, EVO2: Emmetropia Verifying Optical 2.0, Hill3: Hill-RBF 3.0, LSF: Ladas Super formula, MAE: mean absolute refractive prediction error, MedAE: median absolute prediction error, MPE: mean numerical prediction error, SD: standard deviation, Seg AL: segmented axial length, SRK/T: Sanders–Retzlaff–Kraff trial. * Significant difference between the outcomes using composite axial lengths and segmented axial lengths (*p* < 0.05).

## Data Availability

The data presented are available upon request to the corresponding author. The data are not publicly available due to patient privacy.

## Supplementary Materials

The following supporting information can be downloaded at: https://www.mdpi.com/article/10.3390/jcm12226959/s1, Figure S1: The scatter plots of the prediction errors, when comparing the composite AL to segmented AL across seven intraocular lens calculation formulas. Emmetropia Verifying Optical, R2 = coefficient of determination.
