# Supplementary material for: Effect of Segmented Optical Axial Length on the Performance of New-Generation Intraocular Lens Power Calculation Formulas in Extremely Long Eyes
Source: J Clin Med. 2023 Nov 7;12(22):6959. doi: 10.3390/jcm12226959 (PMC10672648; doi:10.3390/jcm12226959)
Supplement: Supplementary file 1 [file jcm-12-06959-s001.zip › jcm-2656856-supplementary.pdf]

Journal of
Clinical Medicine

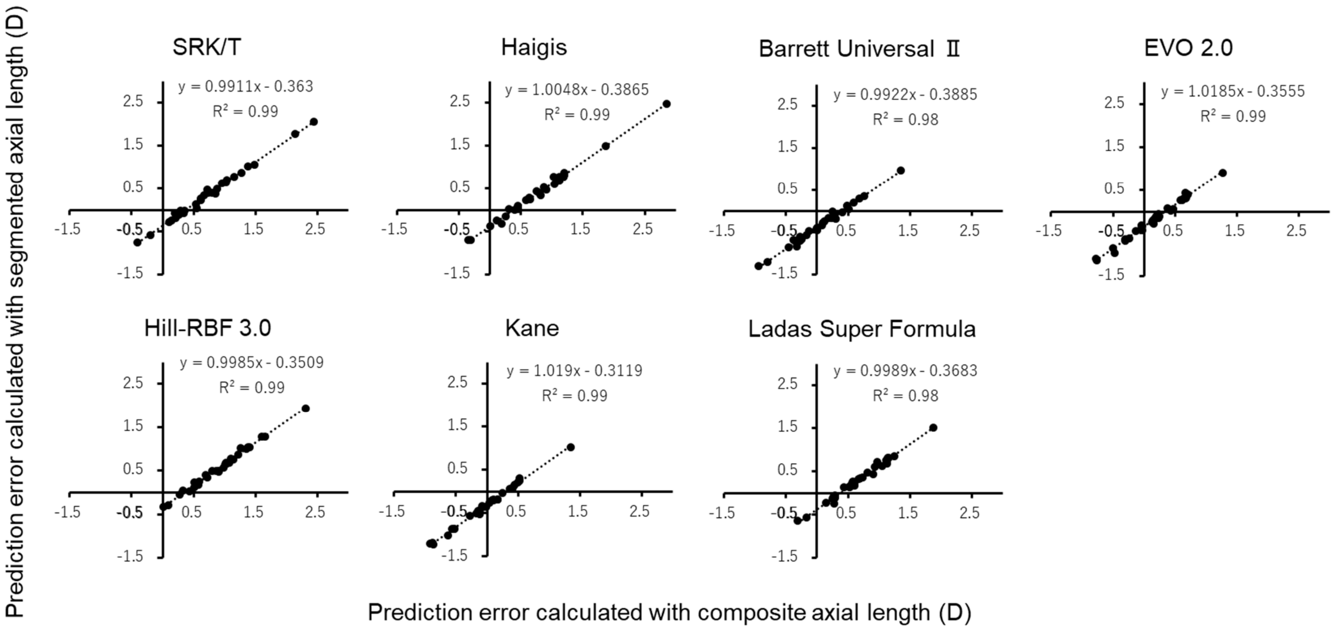

Figure S1. The scatter plots of the prediction errors, when comparing the composite AL to segmented AL across seven intraocular lens calculation formulas. Emmetropia Verifying Optical, R2 = coefficient of determination.
